# Pharmacokinetic data of synthetic cathinones in female Sprague-Dawley rats

**DOI:** 10.1016/j.dib.2018.10.073

**Published:** 2018-10-25

**Authors:** Gregory G. Grecco, David F. Kisor, Jon E. Sprague

**Affiliations:** a The Ohio Attorney General׳s Center for the Future of Forensic Science, Bowling Green State University, Bowling Green, OH 43403, USA; b Indiana University School of Medicine, Indianapolis, IN 46202, USA; c Department of Pharmaceutical Sciences, College of Pharmacy, Natural and Health Sciences, Manchester University, Fort Wayne, IN 46845, USA

**Keywords:** MDMA, 3,4-methylenedioxymethamphetamine, JVC, Jugular Vein Cannula, SMBS, Sodium metabisulfite, EDTA, Ethylenediaminetetraacetic acid, sc, subcutaneous, *C*_max_, maximum concentration, *T*_max_, time of occurrence of maximum concentration, AUC_0−∞_, area under the concentration versus time curve, Vd, volume of distribution, CLp, plasma clearance

## Abstract

The synthetic cathinones methylone, butylone, and pentylone differ from each other through the one carbon lengthening of the α-alkyl chain: methylone (-CH3), butylone (-CH2CH3), and pentylone (-CH2CH2CH3) while 3,4-methylenedioxymethamphetamine (MDMA) differs from methylone by a single oxygen atom. Studies with MDMA, suggests that there may be male and female pharmacokinetic and pharmacodynamic differences. In the present study, we present the plasma pharmacokinetic data relative to a 20 mg/kg, subcutaneous doses of methylone, butylone and pentylone in female Sprague-Dawley rats. Briefly, plasma samples were collected via a jugular vein cannula, purified, and analyzed using a HPLC system. While we have previously reported on the consistent relationship between structure and pharmacokinetics of these synthetic cathinones in male, Sprague-Dawley rats (Grecco and Sprague, 2016), this data set suggests that there is no consistent relationship of chemical structure and pharmacokinetics of methylone, butylone and pentylone in female Sprague-Dawley rats. The findings from the present study further emphasize the need for the inclusion of female subjects in the pharmacokinetic studies of synthetic cathinones as it is very possible male-female differences may exist in rodent models.

**Specifications table**

**Table:** 

**Subject area**	Pharmacology
**More specific subject area**	Pharmacokinetics of Drugs of Abuse
**Type of data**	Table and Figures
**How data was acquired**	A Shimadzu high performance liquid chromatography (Kyoto, Japan) coupled with an autosampler (model SIL-20AC HT) and diode array detector (model SPD-M20A)
**Data format**	Processed and Analyzed
**Experimental factors**	Plasma samples were taken following subcutaneous drug injection, purified, and injected into HPLC to determine drug concentration over time.
**Experimental features**	Very brief experimental description
**Data source location**	Bowling Green, Ohio, U.S.
**Data accessibility**	Data is present in current article.
**Related research article**	Grecco GG, Kisor DF, Magura, JS, & Sprague JE (2017). Impact of common clandestine structural modifications on synthetic cathinone “bath salt” pharmacokinetics. Toxicology and Applied Pharmacology, 328, 18–24.

**Value of the data**

• These sex differences in the toxicology and pharmacokinetics of MDMA may extend to synthetic cathinone which are β-keto analogs of MDMA, such as methylone, butylone, and pentylone (Fig 1), but these sex differences have not been empirically determined.
• The current data set provides the first estimates of pharmacokinetic parameter values for methylone, butylone and pentylone in female Sprague-Dawley rats.
• Compared to previous pharmacokinetic studies with methylone, butylone and pentylone in male Sprague-Dawley rats [2], [6], [7], females demonstrate similar *T*_max_, *C*_max_, and AUC_0−∞_. However, the other pharmacokinetic values we assessed in the females did display differences from males.
• The findings from the present study further emphasize the need for the inclusion of female subjects in the pharmacokinetic studies of synthetic cathinones as it is very possible male-female differences may exist in rodent models.

## 1 Data

The data in Fig. 2 illustrates the plasma concentration versus time profiles for the three synthetic cathinones (20 mg/kg sc.). Pharmacokinetic parameter values determined from synthetic cathinone plasma concentration versus time data are shown in Table 1. For methylone, butylone, and pentylone, *C*_max_ were observed at the 30-min time point (*T*_max_). Pentylone, the most lipophilic drug, demonstrated the highest *C*_max_ at 5252.55 ± 130.5 μg/L and an AUC_0−∞_ of 464,469 + 37,307 μg/L × min which were significantly higher than butylone and methylone (*p* < 0.001 and *p* < 0.05, respectively). The removal of one or two methyl groups from pentylone (butylone or methylone, respectively) represents a greater than five-fold decrease in *C*_max_ and a nearly three-fold decrease AUC_0−∞_. However, no significant differences in *C*_max_ or AUC_0−∞_ were observed when examining methylone and butylone alone.

**Table 1:** Pharmacokinetic estimates of synthetic cathinones in the plasma after subcutaneous administration (20 mg/kg) in female Sprague-Dawley rats (*n* = 4). Parameters were calculated from plasma concentration versus time plots depicted in Fig. 2 with noncompartmental analysis.

Plasma pharmacokinetics			
Drug	Methylone	Butylone	Pentylone
*T*_max_ (min) (median)	30	30	30
*C*_max_ (μg/L)	999.04 ± 56.72	797.72 ± 93.14	5252.55 ± 130.5***
AUC_0__−∞_ (μg/L × min)	165,005 ± 7592*	75,750 ± 8948*	464,469 ± 37,307*
*t*_1/2_ elimination (min)	133.5 ± 18.3*	50.3 ± 8.3	76.6 ± 8.3
CL_p_/F (mL/min)	33.9 ± 2.1*	77.9 ± 9.8*	12.3 ± 1.2*
Vd (mL)	6459.4 ± 824.9	5354.2 ± 361.7	1319.2 ± 57.5**

Data are mean ± S.E.M.

* Indicates significantly different than all other drugs for the pharmacokinetic parameter in that row (*p* < 0.05).

** Indicates significantly different than all other drugs for the pharmacokinetic parameter in that row (*p* < 0.01).

*** Indicates significantly different than all other drugs for the pharmacokinetic parameter in that row (*p* < 0.001).

**Fig. 1:**
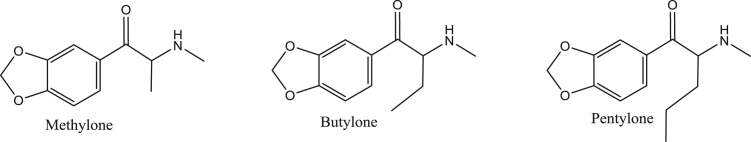
Chemical structures of the synthetic cathinones examined in this study. These synthetic cathinones differ by lengthening of the α-alkyl chain: methylone (-CH_3_), butylone (-CH_2_CH_3_), and pentylone (-CH_2_CH_2_CH_3_).

**Fig. 2:**
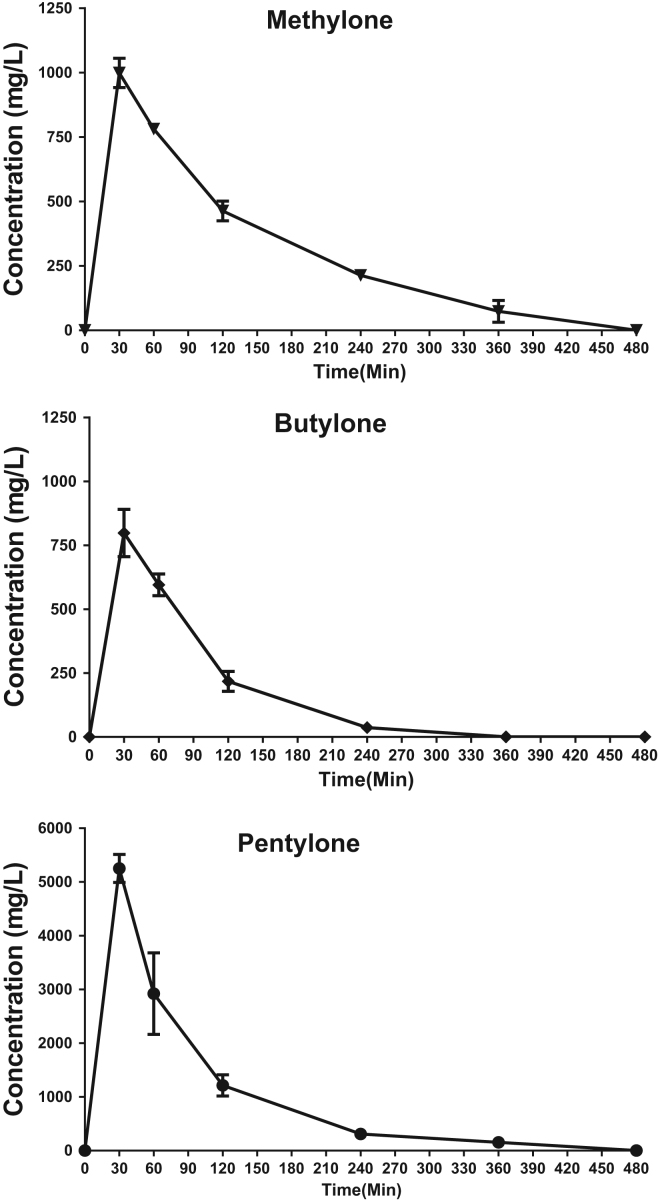
Plasma concentration versus time plots for synthetic cathinones after subcutaneous administration (20 mg/kg). Female Sprague-Dawley rats (*n* = 4) received synthetic cathinone at time 0. Data are expressed as mean ± S.E.M.

Methylone displayed a *t*_1/2_ of 133.5 ± 18.3 min that was significantly longer than butylone or pentylone (*p* < 0.05). The α-alkyl chain lengthening from methylone to butylone and pentylone significantly decreased the CLp/F with pentylone displaying the lowest CLp/F of 12.3 + 1.2 mL/min.

## 2 Experimental design, materials, and methods

### 2.1 Animals

Jugular vein cannulated (JVC) female Sprague-Dawley rats (*n* = 4; 281.75 ± 8.34 g) were obtained from Envigo (Indianapolis, IN)). Animals were housed one per cage (cage size: 21.0 × 41.9 × 20.3 cm) at 24–25 °C, maintained on a 12:12 h light/dark schedule and provided adlibitum access to food and water. Animal maintenance and research were conducted in accordance with the eighth edition of the Guide for the Care and Use of Laboratory Animals as adopted and promulgated by the National Institutes of Health. Protocols were approved by the Bowling Green State University Animal Care and Use Committee.

### 2.2 Plasma pharmacokinetics study design

We utilized a crossover design following a 7-day washout period after which animals were treated with the next synthetic cathinone at 20 mg/kg, subcutaneous (sc.) based on previous pharmacokinetic and pharmacodynamic studies with these agents [5], [6]. Blood samples (250 μL) were collected at time 0, 30, 60, 120, 240, 360, and 480 min post-treatment. Vaginal lavage was performed prior to treatment to confirm estrous cycle phase using a procedure and criterion outlined elsewhere [4]. At the time of treatment, all animals were determined to be in the diestrus-estrus phase. The plasma purification methods were modified from a previous synthetic cathinone plasma purification method [1]. Briefly, the blood samples were centrifuged for 5 min at 4100×*g* to collect plasma layer and formed elements were discarded. Twenty microliters of 250 mM sodium metabisulfite (SMBS) and 10 μL of 250 mM Ethylenediaminetetraacetic acid (EDTA) were added to 100 μL of rat plasma and gently vortexed. Protein precipitation was performed with 400 μL chilled acetonitrile followed by centrifugation at 9800×*g* for 10 min. The supernatant was removed and evaporated to dryness under a stream of nitrogen gas in a 40 °C water bath. The remaining solute was reconstituted in mobile phase A (0.1% formic acid in water). The solution was vortexed and centrifuged at 4100×*g* for 5 min. The supernatant was then transferred to 1.5 mL autosampler vials containing 0.2 mL glass inserts for injection into HPLC. The synthetic cathinone percent extraction recoveries from these methods varied from 70% to 76%.

### 2.3 High performance liquid chromatography

A Shimadzu high performance liquid chromatography (Kyoto, Japan) coupled with anautosampler (model SIL-20AC HT) and diode array detector (model SPD-M20A) system was used for quantifying synthetic cathinone concentrations. Chromatographical separation was achieved with a liquid chromatograph (model LC-20AD) using a Kinetex LC column 150 × 4.6 mm. The gradient elution was performed with mobile phase A (0.1% formic acid in water) and B (0.1% formic acid in acetonitrile) at 0.4 mL/min and 40 °C. Synthetic cathinone spiked standards have shown that different percentages and intervals of mobile phase A and B are required for adequate separation of methylone, butylone, and pentylone. For methylone, mobile phase B was maintained for 0.2 min at 10% and increased to 95% over 3 min. Mobile phase B was then held at 95% for 1 min and returned to 10% over 0.5 min. There was a 2 min equilibrium period yielding a total run time of 7 min. For butylone and pentylone, mobile phase B was maintained for 0.2 min at 90% and decreased to 25% over 3 min. Mobile phase B was then held at 25% for 3 min and lowered to 10% over 1 min. There was a 0.5 min equilibrium period yielding a total run time of 7.5 min. All samples were viewed at a wavelength of 290 nm. The drug concentrations of the plasma and CSF samples were calculated using peak-area ratios from standard solutions spiked with synthetic cathinones. The limit of detection for all synthetic cathinones was determined to be 48.83 ug/L.

### 2.4 Chemicals and reagents

Racemic methylone, butylone and pentylone were obtained as hydrochloride salts from Cayman Chemical (Ann Arbor, MI). All other reagents were obtained from Fisher Scientific (Hampton, NH). On the day of the study, all drug solutions were made fresh at a concentration of 20 mg/mL in normal saline.

### 2.5 Pharmacokinetic and statistical analysis

Synthetic cathinone plasma concentration versus time plots (rectangular and semilogarithmic) were constructed, and the data was analyzed using noncompartmental methods using an industry standard software program (WinNonlin Professional 5.1; Pharsight Co., Mountain View, CA) to estimate pharmacokinetic parameter values. The plasma *C*_max_ was the highest observed plasma concentration and the time of the occurrence of the *C*_max_ (*T*_max_) was also observed. The terminal elimination rate constant (*λz*) was calculated via linear regression of the observed terminal natural log concentration versus time data and *t*_1/2_ was calculated as 0.693/*λz*. At least three concentration-time points were used to determine *λz*. The volume of distribution (VD) was calculated by the method described by Gibaldi and Perrier [3]. Descriptive pharmacokinetic data is presented as the mean ± S.E.M. (with exception of the *T*_max_ which is given as the median value) for each group of animals for each synthetic cathinone. The AUC_0−∞_ represents the total area under the plasma concentration-time curve from time zero to infinity, and was calculated using the linear trapezoidal rule with the terminal AUC being calculated as the last measured concentration divided by *λz* (Clast/*λz*). VD was calculated as a ratio of the total dose present in the body to the plasma concentration when the distribution of the drug between the tissues and the plasma is at an equilibrium. The CLp/F corresponds to the plasma clearance of 9 the drug being calculated as dose/AUC_0−∞_ considering extravascular dosing. GraphPad InStat v.6.0 software was used to complete all statistical analyses of data. Synthetic cathinone pharmacokinetic values were compared using one-way ANOVA with the test drug as the main factor, and in the presence of a significant main effect, a Student–Newman–Keuls post-hoc test was conducted. When only two groups were compared, a Student׳s *t*-test was used. Significance was set a priori at the 95% confidence level (*p* < 0.05).
